# Online Monitoring of the Growth of Probiotic Bacteria and Metabolites in the Fermentation of a Teff Substrate Using Model-Based Calibration of 2D Fluorescence Spectra

**DOI:** 10.3390/microorganisms11041032

**Published:** 2023-04-15

**Authors:** Sendeku Takele Alemneh, Majharulislam Babor, Viktoria Zettel, Almut von Wrochem, Bernd Hitzmann

**Affiliations:** Department of Process Analytics and Cereal Science, Institute of Food Science and Biotechnology, University of Hohenheim, 70599 Stuttgart, Germany; majhar@uni-hohenheim.de (M.B.); viktoria.zettel@uni-hohenheim.de (V.Z.); almut.vonwrochem@uni-hohenheim.de (A.v.W.)

**Keywords:** fermentation process, fluorescence spectroscopy, probiotic bacteria, teff

## Abstract

The demand for probiotic bacteria-fermented food products is increasing; however, the monitoring of the fermentation process is still challenging when using conventional approaches. A classical approach requires a large amount of offline data to calibrate a chemometric model using fluorescence spectra. Fluorescence spectra provide a wide range of online information during the process of cultivation, but they require a large amount of offline data (which involves laborious work) for the calibration procedure when using a classical approach. In this study, an alternative model-based calibration approach was used to predict biomass (the growth of *Lactiplantibacillus plantarum* A6 (LPA6) and *Lacticaseibacillus rhamnosus* GG (LCGG)), glucose, and lactic acid during the fermentation process of a teff-based substrate inoculated with mixed strains of LPA6 and LCGG. A classical approach was also applied and compared to the model-based calibration approach. In the model-based calibration approach, two-dimensional (2D) fluorescence spectra and offline substituted simulated data were used to generate a chemometric model. The optimum microbial specific growth rate and chemometric model parameters were obtained simultaneously using a particle swarm optimization algorithm. The prediction errors for biomass, glucose, and lactic acid concentrations were measured between 6.1 and 10.5%; the minimum error value was related to the prediction of biomass and the maximum one was related to the prediction of glucose using the model-based calibration approach. The model-based calibration approach and the classical approach showed similar results. In conclusion, the findings showed that a model-based calibration approach could be used to monitor the process state variables (i.e., biomass, glucose, and lactic acid) online in the fermentation process of a teff-based substrate inoculated with mixed strains of LPA6 and LCGG. However, glucose prediction showed a high error value.

## 1. Introduction

Modern consumers’ interest in healthy diets is increasing with the rise in their awareness about the association between food and health. Probiotic fermented food products, with an emphasis on cereal-based probiotic foods, are an alternative means of meeting consumers’ current food preferences. In particular, non-dairy probiotic fermented food products are in demand because of the increasing numbers of consumers embracing vegetarianism due to medical reasons and personal preferences, as well as the drawbacks associated with dairy-based products [1]. It is necessary to control the fermentation process to determine important state variables in order to produce a satisfactory food product. However, monitoring of the fermentation process is usually challenging when using classical approaches, which are time consuming, costly, and labor intensive [1,2]. 

In the fermentation process, small changes can influence the quality of the final product; therefore, it is important to pay attention to controlling undesirable outcomes on time during the fermentation process [3]. Fluorescence spectroscopy is one of the methods of interest in monitoring and supervising bioprocess systems; however, a classical spectroscopic-based monitoring system often requires a large amount of offline data to generate a chemometric model and to predict the state variables [4]. Recently, 2D fluorescence spectroscopy has become a good alternative means of monitoring biological systems, since it allows us to collect information about the physiological state of the organisms [5]. Additionally, it has become an increasingly well-known system for the online monitoring of bioprocesses, as it allows for the measurement of many metabolic substances simultaneously [6]. Furthermore, 2D fluorescence spectroscopy gathers information in a non-invasive way as it does not interfere with the state of the bioprocesses. Clearly, a wide range of spectrum data are obtained during a fluorescence spectroscopy measurement, which therefore requires techniques for the extraction of important information. For the evaluation of spectrum data, chemometric methods such as principal component regression and partial least squares regression can be applied [2,7]. 

The usual chemometric model calibration process requires a significant amount of offline data to develop an accurate model, which is the key limitation of the classical approach. However, a model-based calibration approach may be a good alternative, as it does not require a time-consuming, labor-intensive, and expensive method for the collection of results [8]. In a model-based calibration approach, important information can be obtained from prior knowledge about the process: for instance, a mathematical model, whose parameters can be estimated in the calibration procedure. Extracted information related to the process variables can be used for model-based calibration [9]. 

For the purpose of reducing the number of offline measurements, a mechanistic process model could be fitted to the online measurement system. However, in this approach, the parameters of the process model, such as the specific growth rate of the microbes and the yield, have to be determined, which depends on the bioprocess that is under investigation. This can be obtained by fitting a process model with offline measurements from an earlier run of the same system. Once the process parameters are known, the process model can be used to simulate the actual state of the bioprocess. Then, for the calibration of a chemometric model, no further offline data are required. Instead, simulated data using the process model serve as the offline data. Of course, some offline results are still required to fit the mechanistic process model to reflect the actual state of the bioprocess. However, the total number of offline data are minimized and are used only once [6]. 

A similar approach was adopted by Solle et al. [9]; simulated data collected from a theoretical process model coupled with the corresponding fluorescence spectra were applied to calibrate a chemometric model for the online monitoring of *Saccharomyces cerevisiae* and to find the process model parameters. A cultivation process was simulated using different values of the specific growth rate and a chemometric model was calibrated using the simulated data and fluorescence spectra. When using this approach, the best chemometric model can be achieved when the process parameters used for the process simulation and the actual parameters of the bioprocess are equivalent. A model-based calibration approach was described by Babor et al. [10] to monitor the state of *Hansenula polymorpha* cultivation online, where chemometric models were calibrated only based on 2D fluorescence spectra. The study showed promising results in predicting biomass and glycerol with good accuracy. 

Regardless of this, information was not found in literature about the application of a model-based calibration approach in relation to the fermentation process of a teff substrate inoculated with probiotic lactic acid bacteria. Therefore, in this study, a chemometric model was calibrated using 2D fluorescence spectra and simulated data obtained from a theoretical process model of LPA6 and LCGG cultivation. This calibrated chemometric model could be used for the online prediction of the growth of probiotic bacteria, as well as concentrations of glucose and lactic acid, in the process of fermenting a teff-based substrate inoculated with LPA6 and LCGG. 

## 2. Materials and Methods

### 2.1. Materials

Wholegrain teff flour was purchased from Teff-shop.de, Manuel Boesel, Homburger Str. 49a, 61191 Rosbach von der Höhe, Germany. Freeze-dried microorganisms, *Lactiplantibacillus plantarum* A6 (LMG 18053, BCCM, Gent, Belgium), and *Lacticaseibacillus rhamnosus* GG (LMG, 18243, BCCM, Gent, Belgium) were bought from Belgium. 

### 2.2. Starter Culture Preparation 

Starter culture strains of LPA6 and LCGG were prepared using the same method used by Alemneh et al. [11] and were put in a refrigerator until utilization at 48 h. A starter culture of LPA6 was obtained by overnight incubation in an incubator (BINDER GmbH, KB 115, Tuttlingen, Germany) at 30 °C in sterile MRS broth; meanwhile, the starter culture of LCGG was obtained by overnight incubation at 37 °C in sterile MRS broth. For inoculation, starter cultures were harvested by centrifugation (Mega star 600R, Leuven, Belgium) at 3000× *g*, 4 °C, for 15 min. Cell pellets were washed with a sterile saline solution (0.9% NaCl) and centrifuged again. Then, the supernatant was removed and cell pellets were re-suspended in a saline solution to form a cell suspension of about 9 log CFU/mL (CFU stands for colony-forming units) and taken as the inoculum. 

### 2.3. Enumeration of Viable Microbes and Fermentation Conditions

The total cell counts of LPA6 and LCGG were measured using plate count agar according to the method used by Alemneh et al. [12]. Approximately 15 g agar was mixed in 1 L MRS broth to prepare MRS agar (Carl Roth GmbH + Co. KG, Karlsruhe, Germany). Ten-fold serial diluted samples were prepared with saline solution. Then, about 50 µL of the samples were put onto MRS agar plates and incubated for 48 h at 30 °C to find the cell counts of LPA6 and LCGG. 

Mixed-culture strains of LPA6 and LCGG with inoculum levels of 6 and 5 log CFU/mL, respectively, were inoculated to the fermentation medium. The fermentation medium was prepared from 7 and 4 g wholegrain teff flour in 100 mL distilled water. Three fermentation runs were performed each with different initial condition. The initial conditions were (1) 7 g teff flour in 100 mL distilled water inoculated with 6 log CFU/mL of LPA6 and LCGG, (2) 4 g teff flour in 100 mL distilled water inoculated with 6 log CFU/mL of LPA6 and LCGG, and (3) 7 g teff flour in 100 mL distilled water inoculated with 5 log CFU/mL of LPA6 and LCGG. Before fermentation, the substrate was heated in a water bath (GFL-1083, Bugwedel, Germany) set at 85 °C for 15 min and was then sterilized (SHP Laboklav, 160-MSLV, Satuelle, Germany). The substrate was cooled down before the inoculation of LPA6 and LCGG. Finally, fermentation was performed using a 2.5 L Bioreactor (INFORS AG CH-4103, Bottmingen, Switzerland) by stirring at 150 rpm without pH control for 15 h at 37 °C. For the chemometric model calibration, 2D fluorescence data were collected from the three different fermentation runs. Each chemometric model was calibrated based on spectra from two cultivations and validated using the spectra obtained from the other fermentation run. The process was repeated until every cultivation was used once for validation. 

### 2.4. Offline and Online Measurements 

For the offline measurements of glucose and lactic acid, high-performance liquid chromatography (HPLC) was used. Samples were centrifuged at 3000× *g*, 4 °C for 15 min and the supernatant was filtered with a 0.45 μm polypropylene membrane (VWR, Darmstadt, Germany). Then, the sample supernatant was quantified with an HPLC (ProStar, Variant, Walnut Creek, CA, USA), which was equipped with a refractive index detector. About 20 μL of the sample was injected into a Rezex ROA-organic acid H+ (8%) column (Phenomenex, Aschaffenburg, Germany) operated at 70 °C. A 5 mM H_2_SO_4_ solvent with a flow rate of 0.6 mL/min was used. The quantities of analytes were calculated using the chromatography software GalaxieTM, version number 1.10.0.5590 (Varian, Walnut Creek, CA, USA). 

For the online measurements, 2D fluorescence spectra were collected using a BioView sensor (DELTA Lights & Optics, Venlighedsvej 4, 2970, Horsholm, Denmark). The fluorescence probe was connected to the sterilized bioreactor over a light guide linked to a 25 mm standard port, which contained a quartz glass window to interface with the bioreactor. Therefore, there was no contact between the fermentation medium and the actual sensor tip. The sensor measured multi-wavelength fluorescence in the ranges of 270–550 nm excitation and 310–590 nm emission. A resulting spectrum consisted of 120 intensity values of wavelength combinations measured in steps of 20 nm. 

### 2.5. Process Simulation and Optimization 

Simulation of process models and optimizations were performed using the computer language MATLAB R2022a on a computer with a configuration of an Intel i5 at 4 × 3.20 GHz, with 8 GB ram, running Microsoft window 10. 

### 2.6. Process Simulation Models

Simulation of the bioprocess with a theoretical model is an important technique in the fields of bioprocess applications [13]. In previous work, when a substrate made of wholegrain teff flour was fermented with mixed strains of LPA6 and LCGG, glucose and lactic acid were the main metabolites consumed and produced, respectively [11]. Hence, in this study, the calibrated model was applied to predict the growth of LPA6 and LCGG and the metabolite concentrations of glucose and lactic acid. The cultivation processes of LPA6 and LCGG can be simulated using the following equations (Equations (1)–(5)):

(1) $${\;X}_{t}=X_{o}e^{µt}$$

(2) $$G_{t}=G_{o}-\frac{X_{o}}{Y_{\mathrm{GX}}}e^{µt}+\frac{X_{o}}{Y_{\mathrm{GX}}}$$

(3) $${\;L}_{t}=\frac{X_{o}}{Y_{\mathrm{GL}}}e^{µt}-\frac{X_{o}}{Y_{\mathrm{GL}}}$$

subject to:

(4) $$G_{t}\geq 0$$

(5) $$X_{t}=\left\{\begin{array}{c} X_{t}\;\;\;\;if\;G_{t}>0\\ X_{t-1}\;if\;G_{t}=0\; \end{array}\right.$$

where *X_t_* is the biomass (cell counts of LPA6 and LCGG), µ is the microbial specific growth rate, X_o_ is the inoculated biomass, t is the fermentation time, *G_t_* is the glucose concentration, G_o_ is the initial glucose concentration, Y_GX_ is the yield coefficient with respect to the conversion from glucose to biomass, L_t_ is the lactic acid concentration, and Y_GL_ indicates the yield coefficient with respect to the conversion from glucose to lactic acid. 

### 2.7. Classical Approach to the Optimization of Process Model Parameters 

To describe the initial phase, the process was simulated with random process parameters such as the specific growth rate (µ), the yield coefficient with respect to the conversion from glucose to biomass (Y_GX_), and the yield coefficient with respect to the conversion from glucose to lactic acid (Y_GL_). However, the concentration of the state variables, such as biomass, glucose, and lactic acid from simulation, might not reflect their actual concentrations. Since the process parameters are specific to a given bioprocess, the first step was to determine the process parameters. To find the optimum process model parameters (µ, Y_GX,_ and Y_GL_) of the bioprocess under consideration, simulated process state variables were used to fit against the previously obtained offline data. The particle swarm optimization algorithm [14] was used to find the optimum process parameters, for which the simulated state variables fit to the corresponding offline data. 

In the fitting criterion, the objective of the optimization was to minimize the sum of squared difference in the concentration of state variables between the simulated and the corresponding offline values. For each variable, one fitness value is calculated, which were then normalized to ensure their equal impact on fitness criterion. For any chosen combination of process parameters (µ, Y_GX,_ Y_GL_), if the sum of normalized fitness values is higher, it indicates that the simulated concentrations are significantly deviated from the actual measurements. A solution from particle swarm optimization is an optimum combination of process parameters (µ, Y_GX,_ Y_GL_), for which the sum of the normalized fitness values is the minimum. The process model can estimate the state variables at any measurement point using the optimized process parameters with the initial conditions. More specifically, a classical approach for the optimization of process parameters is an optimized problem solved iteratively to find the optimum combination of process parameters, for which the simulated values are made equivalent to the offline values by using a least-square fitting approach. The quality functions are presented in Equations (6)–(9):

(6) $$\mathrm{SE}=G_{\mathrm{RMSE}}+L_{\mathrm{RMSE}}+X_{\mathrm{RMSE}}$$

(7) $$G_{\mathrm{RMSE}}=\sqrt{\frac{\sum_{i=1}^{n}{\left(G_{i}^{0\mathrm{ff}}-G_{i}^{\mathrm{sim}}\right)}^{2}}{n}}$$

(8) $$L_{\mathrm{RMSE}}=\sqrt{\frac{\sum_{i=1}^{n}{\left(L_{i}^{0\mathrm{ff}}-L_{i}^{\mathrm{sim}}\right)}^{2}}{n}}$$

(9) $$X_{\mathrm{RMSE}}=\sqrt{\frac{\sum_{i=1}^{n}{\left(X_{i}^{0\mathrm{ff}}-X_{i}^{\mathrm{sim}}\right)}^{2}}{n}}$$

where SE is the sum of error,${\;G}_{\mathrm{RMSE}}$, $L_{\mathrm{RMSE}}$, and $X_{\mathrm{RMSE}}$ are the root mean squared errors for glucose, lactic acid, and biomass, respectively, i is the measurement index, n is the number of observations, $G_{i}^{0\mathrm{ff}}$, $L_{i}^{0\mathrm{ff}},$ and $X_{i}^{0\mathrm{ff}}$ are offline glucose, lactic acid, and biomass, respectively, and $G_{i}^{\mathrm{sim}}$, $L_{i}^{\mathrm{sim}},$ and $X_{i}^{\mathrm{sim}}$ are simulated glucose, lactic acid, and biomass, respectively. 

**Figure 1:**
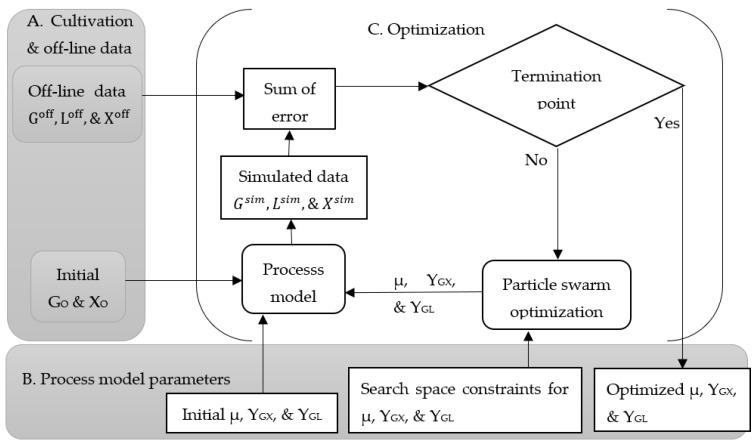
Classical approach for the optimization of the process model parameters for the cultivation of *Lactiplantibacillus plantarum* A6 and *Lacticaseibacillus rhamnosus* GG; G_O_ and X_O_ are the initial concentrations of glucose and biomass, respectively; G^off^, L^off^, and X^off^ are the offline data for glucose, lactic acid, and biomass, respectively; G^sim^, L^sim^, and X^sim^ are the simulated data for glucose, lactic acid, and biomass, respectively; µ, microbial specific growth rate; Y_GX_, yield coefficient with respect to the conversion from glucose to biomass; and Y_GL_, yield coefficient with respect to the conversion from glucose to lactic acid. (**A**) displays the offline data acquired during cultivation, (**B**) shows the initial and optimized process model parameters, and (**C**) illustrates the optimization process.

Figure 1 shows how the classical approach proceeds to optimize the process parameters using offline data and a mathematical process model. This approach starts with the initial concentrations of biomass (X_O_) and glucose (G_O_) with a random initial set of process parameters: specific growth rate (µ), yield coefficient with respect to the conversion from glucose to biomass (Y_GX_), and yield coefficient with respect to the conversion from glucose to lactic acid (Y_GL_). The constraints of the process parameters for the search space are defined based on initial investigations and set to a range of 0.2–0.9 [10^8^ CFU/h] for µ and 2.0–4.0 (10^8^ CFU/g) for Y_GX_ and Y_GL_.

### 2.8. Model-Based Calibration Approach for the Optimization of the Process Model and Chemometric Model Parameters 

In this section, a model-based calibration approach was performed without offline data. Instead, simulated data obtained from the process model served as the offline data. Simulated data calculated with the process model and the actual 2D fluorescence spectra were mapped with respect to the cultivation time to calibrate the chemometric model. Simulated data were taken as target values and 2D fluorescence spectra as the independent variable to articulate the chemometric model for the prediction of state variables (biomass, glucose, and lactic acid). When using this approach, the optimized microbial specific growth rate (µ) and the optimized chemometric parameters were obtained simultaneously. In addition to the microbial specific growth rate (µ), the yield coefficients ($Y_{\mathrm{GX}}$ and $Y_{\mathrm{GL}}$) are required to simulate the cultivation process. In this procedure, the yield coefficients were roughly guessed based on previous experience and were kept constant. Spectra data were divided into the training and test sets, and the training spectra and the corresponding simulated data were used to calibrate the chemometric model. With this calibrated chemometric model, the prediction of the simulated test set values was executed. According to the description given in [10], the predicted simulated test values and simulated test values might show large differences, since the features extracted from actual spectra might not correlate with the simulated values. 

**Figure 2:**
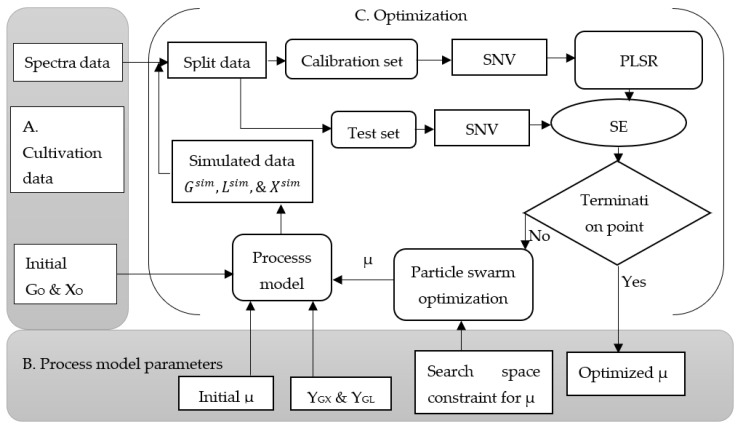
Model-based calibration process to find the optimized parameters in the cultivation of *Lactiplantibacillus plantarum* A6 and *Lacticaseibacillus rhamnosus* GG; G_O_ and X_O_ are the initial concentrations of glucose and biomass, respectively; G^off^, L^off^, and X^off^ are the offline data for glucose, lactic acid, and biomass, respectively; G^sim^, L^sim^, and X^sim^ are the simulated data for glucose, lactic acid, and biomass, respectively; µ, microbial specific growth rate; Y_GX_, yield coefficient with respect to the conversion from glucose to biomass; Y_GL_, yield coefficient with respect to the conversion from glucose to lactic acid; SNV is the standard normal variate; SE is the sum of the error; and PLSR is the partial least square regression. (**A**) shows spectra data and initial values of biomass and glucose; section (**B**) serves as input for the main optimization cycle carried out in section (**C**) with the initial parameters for the theoretical process model and the corresponding search space costraints.

Figure 2 shows the model-based calibration procedure. Using this approach, a partial least square regression model of 2D fluorescence spectra was performed. Three components with high variances were taken for the prediction of biomass, but five components were considered for the predictions of glucose and lactic acid. The objective was to find the optimum microbial specific growth rate (µ) and chemometric model parameters simultaneously, where the state variables predicted using a chemometric model and simulated using a theoretical process model fitted the best at the point where the sum of the error showed the minimum value. The particle swarm optimization algorithm was used to find the optimum values of these parameters. Here, once the optimum parameters and initial state variables (biomass and glucose) were obtained, chemometric models were able to give the predicted results of the state variables. Additionally, the process model was able to give the simulated data of the corresponding state variables, as shown in Equations (1)–(3). 

The fitness criterion was the sum of the squared difference of the state variables between the simulated values and the corresponding predicted values. A minimum sum of squared difference indicates better fitting, and thus a robust model-based chemometric model is achieved. However, particle swarm optimization requires several iterations to reach to an optimum combination of these parameters for which the error is minimum; in this way, the best possible model-based calibration state is achieved. This procedure is followed for all of the state variables (biomass, glucose, and lactic acid) to produce separate model-based calibrations. 

As shown in Figure 2, a model-based calibration approach is described as follows: a combination of excitation and emissions wavelengths is prepared to remove the scattered light, which is not important for the calibration process. Initial concentrations of biomass (X_O_), glucose (G_O_), and lactic acid (L_O_) are assumed to be known; the constraint of the process parameter for the search space is defined based on initial investigations and set to a range of 0.2–0.9 (10^8^ CFU/h) for µ, but for Y_GX_ and Y_GL_, values were kept constant at 3.4 and 2.5 × 10^8^ cfu/g, respectively. Initially, a random value for the microbial specific growth rate within the search space was proposed; the cultivations were simulated with the mathematical process model by using the proposed growth rate and the initial conditions. Different initial conditions could give different simulated values at a given point of measurement during the cultivation time; the intensity values of the fluorescence spectra were normalized with SNV and a partial least square regression model was applied. 

The simulated concentrations of biomass, glucose, and lactic acid were aligned to the 2D fluorescence spectra with respect to the cultivation time. The obtained data was divided into two as calibration and test sets. Data from two cultivations were considered for calibration and the rest one cultivation was applied for testing models. In this way, always one complete cultivation was used for testing, and this was repeated until each cultivation was tested once (cross-validation). The calibration set contained simulated biomass, glucose, and lactic acid as well as 2D fluorescence spectra from two cultivations, whereas the test set had simulated biomass, glucose, and lactic acid, and fluorescence spectra from the remaining cultivation; a multilinear regression model was fitted using the calibration set and then tested with the test set; particle swarm optimization was used to evaluate the sum of the error and the optimization procedure was used to minimize the error. To improve the prediction error, particle swarm optimization proposed a new value of µ in the search space and it continued until a termination criterion was met. The optimized value of µ was saved at the point where the best predicted values of the state variables was observed. Finally, the observed optimum parameters of the theoretical process model and the chemometric model were used for further validation. 

### 2.9. Validation of Model-Based Calibration

Offline data were used only to validate whether the proposed model-based calibration approach estimated the process variables accurately or not. The complete calibration procedure and the cross-validation were performed using 2D fluorescence spectra and simulated process variables. To examine the performance of these approaches, offline data were used for the comparison of the simulated and predicted data. 

## 3. Results and Discussion

### 3.1. Initial Conditions for the Cultivation and Optimization of the Process Parameters 

Online monitoring of the bacterial probiotic fermentation process is important to controlling the process in real time and producing a satisfactory product. However, the systems for monitoring the fermentation process that use conventional approaches are time consuming, costly, and labor intensive. Therefore, an alternative procedure is necessary to monitor the microbial cultivation process online. For instance, the application of mathematical models may be an option for supervising the fermentation process in real time. Moreover, as described by Balsa-Canto et al. [15], the potential of mathematical models to describe microbial behavior in the fermentation process using classical approaches can be improved by model calibration. Table 1 shows three initial cultivations, two of which were used for calibration, while the remaining cultivation was used for testing purpose.

In this study, biomass represents the viable cell counts of LPA6 and LCGG in CFU/mL. Ideally, it is expected that the optimized process parameters should reflect the system despite having variations in the initial conditions. Therefore, in this study, fermentations with three different combinations of initial conditions were considered. 

In a model-based calibration approach, the simulation model parameters are important factors for the prediction errors. If the results of a simulation model’s parameters are in close agreement with the actual values, then the process model could monitor the cultivation process effectively [8]. Therefore, the optimization of the cultivation process should be performed with great care to obtain results with less uncertainty. 

In this study, for the optimization of important parameters in the fermentation of a teff-based substrate inoculated with bacterial probiotics (LPA6 and LCGG), classical and model-based calibration approaches were utilized. In the classical approach, the process model was fitted with the actual offline data and the particle swarm optimization algorithm was used to find the optimum parameters. However, in the model-based approach, offline data were not considered for the calibration process; instead of the offline data, the simulated data were used. Here, the chemometric models were calibrated using only the theoretical process model and 2D fluorescence spectra collected over the fermentation time of the teff-based substrate inoculated with mixed strains of LPA6 and LCGG. 

Table 2 shows the optimized process parameters obtained using the theoretical process model with two different approaches, i.e., the classical approach and the model-based calibration approach. The difference between the classical and model-based calibration approaches was the type of data compared with the simulated data i.e., in the classical approach, offline data were compared with simulated data and, in the model-based calibration approach, predicted data (data obtained from the prediction of chemometric model) were compared with simulated data (data obtained from the simulation of the theoretical process model).

### 3.2. Validation of the Model-Based Calibration Approach

The performance of the model-based calibration approach was validated using its data; for this purpose, every cultivation was left out of the calibration process once. The calibration process obtained the optimized process parameters and the chemometric models simultaneously for the predictions of biomass, glucose, and lactic acid concentrations. Validation of the model-based calibration approach was carried out if the optimized process parameters represented the left-out cultivation and if the chemometric model could predict process variables from the 2D fluorescence spectra of the left-out cultivation. Therefore, the optimized process parameters with the initial conditions were used to simulate the left-out cultivation, and these simulated values of the left-out cultivation were compared to the actual offline measurements. In the same way, the chemometric models used to predict biomass, glucose, and lactic acid concentrations and this predicted values of biomass, glucose, and lactic acid were compared to the actual offline measurements. For the calculation of the average root mean squared error (RMSE), three repeated validations were performed, and, each time, a different cultivation was left out from the calibration.

A comparison of the simulated biomass, glucose, and lactic acid concentrations, including the actual offline measurements, is presented in Table 3. The simulated biomass, glucose, and lactic acid concentrations were obtained from the process model using the optimized process parameters. The simulated parameters calculated from the model-based calibration approach are close to the parameters calculated by the classical approach, while the model-based calibration approach did not use offline data. Additionally, almost the same errors were observed in both the classical and model-based calibration approaches, as compared to the actual offline measurements. Generally, errors were observed from 6.1 to 10.5% in both the classical and model-based calibration approaches as compared to the actual offline data; here, the maximum error was related to simulated glucose with the classical approach and the minimum error was related to simulated biomass with the model-based calibration approach.

The validation values of the chemometric model predictions are presented in Table 4. The chemometric model validation errors for the concentrations of biomass, glucose, and lactic acid ranged from 6.9 to 10.5%; here, the minimum error is related to the predicted biomass using the classical approach and the maximum error is related to predicted glucose using both the model-based calibration and classical approaches. Similar results were observed in both the classical and model-based calibration approaches, which verifies the that model-based calibration approach is as efficient as the classical approach. In other studies that use the same approach, glycerol showed high validation errors of 8.6% [10] and 10% [6] during the calibration of chemometric models in the cultivation of *Hansenula polymorpha* using 2D fluorescence spectra and theoretical process models.

**Figure 3:**
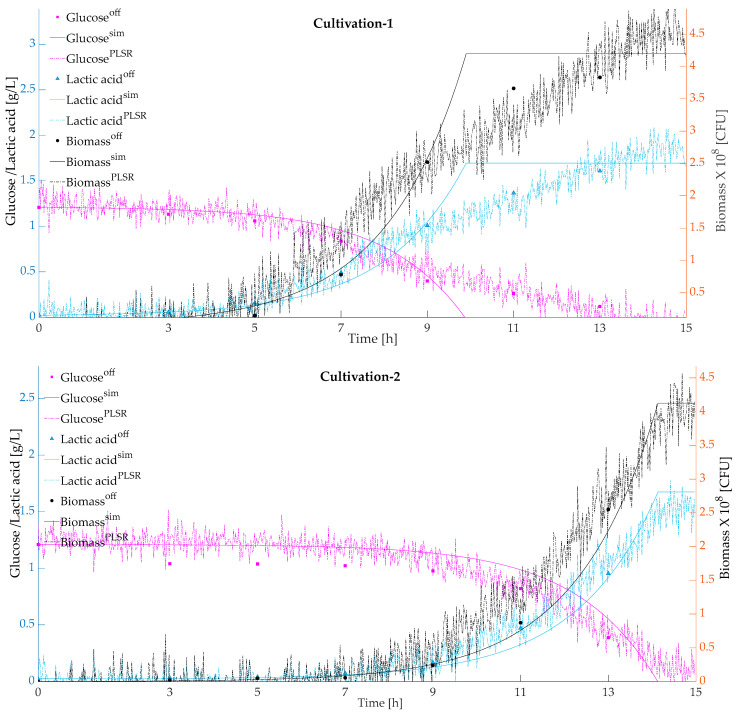

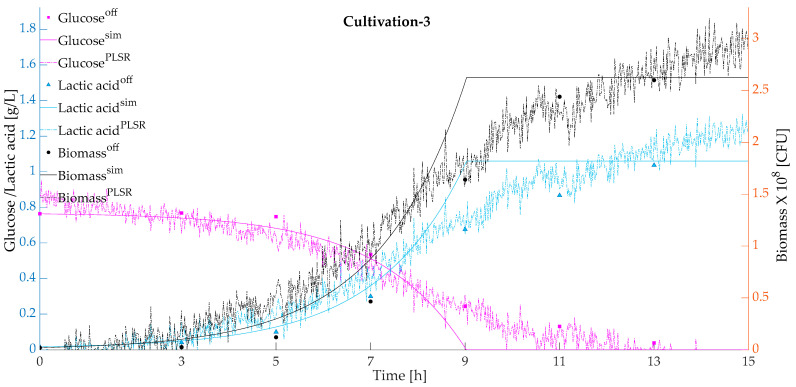
Validation of the chemometric model calibrated from 2D fluorescence spectra using a model-based calibration approach to predict biomass, glucose, and lactic acid, aligned with the actual offline measurements.

Figure 3 presents the predicted and simulated values validated with respect to the actual offline measurements for the cultivations using a model-based calibration approach. Simulated and predicted biomass, glucose, and lactic acid were plotted against the actual offline measurements.

## 4. Conclusions

Bacterial probiotic fermented foods are in demand, and the real-time monitoring of the fermentation process is necessary to produce satisfactory foods. However, monitoring a fermentation process using traditional methods is challenging, since these methods are time consuming, costly, and laborious. For example, large amounts of offline data are required in the traditional approaches for the calibration of a chemometric model. In this work, classical and model-based calibration approaches were used to monitor the cultivation process of a teff-based substrate inoculated with mixed strains of *Lactiplantibacillus plantarum* A6 and *Lacticaseibacillus rhamnosus* GG, which are well-known and studied probiotic bacteria strains. Here, the control target state variables were cell counts of *Lactiplantibacillus plantarum* A6 and *Lacticaseibacillus rhamnosus* GG, including concentrations of glucose and lactic acid. Using the classical approach, the optimized process parameters were obtained by fitting the offline data against the simulated data. The best-fitted simulated data were used for the calibration of the chemometric models and to predict the process variables from 2D fluorescence spectra. Meanwhile, the model-based calibration approach used the simulated data (instead of offline data) from a process model for the calibration process. In the model-based calibration approach, the optimum process parameters and chemometric parameters were obtained simultaneously directly using 2D fluorescence spectra and a process model that describes the cultivation of *Lactiplantibacillus plantarum* and *Lacticaseibacillus rhamnosus*. Hence, the time required for calibration and the difficulty of collecting offline data could be minimized significantly by using this approach. In this approach, offline data were finally used for validation purpose. The findings showed that the model-based calibration approach could be used for the online supervision of process state variables in the fermentation of a teff-based substrate inoculated with mixed strains of *Lactiplantibacillus plantarum* A6 and *Lacticaseibacillus rhamnosus* GG.

## Figures and Tables

**Table 1:** Initial concentrations of glucose and biomass (initial levels of *Lactiplantibacillus plantarum* A6 and *Lacticaseibacillus rhamnosus* GG).

Cultivations	Initial Glucose (g/L)	* Initial Biomass (10^8^ CFU/mL)
Cultivation 1	1.21	0.02
Cultivation 2	1.21	0.002
Cultivation 3	0.76	0.02

* Initially inoculated cell counts of Lactiplantibacillus plantarum A6 and Lacticaseibacillus rhamnosus GG.

**Table 2:** Optimized process parameters calculated with the classical and modal-based calibration approaches.

Approaches	µ (10^8^ CFU/h)	Y_GX_ (10^8^ CFU/g)	Y_GL_ (g/g)
Classical	0.53	3.33	2.68
Model-based calibration	0.54	3.40 *	2.50 *

* The yield factors were kept constant during model-based calibration approach and roughly guessed based on previous cultivation experience; µ, specific growth rate; Y_GX_, yield coefficient with respect to the conversion from glucose to biomass; Y_GL_, yield coefficient with respect to the conversion from glucose to lactic acid.

**Table 3:** Validation of the simulated data with respect to the actual offline data. Simulated biomass, glucose, and lactic acid were used to train the chemometric model and to perform the partial least square regression model.

Approaches	Biomass RMSE		Glucose RMSE		Lactic Acid RMSE	
	$\left(10^{8}\mathrm{CFU}\right)$	(% range)	(g/L)	(% range)	(g/L)	(% range)
Classical	0.33	6.3	0.13	10.5	0.15	7.9
MBC	0.32	6.1	0.13	10.3	0.16	8.3

RMSE, root mean square error; MBC, model-based calibration; biomass refers to the cell counts of *Lactiplantibacillus plantarum* A6 and *Lacticaseibacillus rhamnosus* GG.

**Table 4:** Validation of the predicted biomass, glucose, and lactic acid concentrations with respect to the actual offline measurements. The prediction was performed by the chemometric model (partial least square regression) trained with the simulated concentrations of biomass, glucose, and lactic acid.

Approaches	Biomass RMSE		Glucose RMSE		Lactic Acid RMSE	
	$\left(10^{8}\mathrm{CFU}\right)$	(% range)	(g/L)	(% range)	(g/L)	(% range)
Classical	0.36	6.9	0.13	10.5	0.16	8.6
MBC	0.37	7.1	0.13	10.5	0.15	8.0

RMSE, root mean square error; MBC, model-based calibration; biomass refers to the cell counts of *Lactiplantibacillus plantarum* A6 and *Lacticaseibacillus rhamnosus* GG.

## Data Availability

Not applicable.
